# Effect of High-Flow Nasal Cannula vs. Facemask on Arterial Oxygenation During Liver Radiofrequency Ablation: Randomized Controlled Trial

**DOI:** 10.3390/medicina61071130

**Published:** 2025-06-23

**Authors:** Jung-Pil Yoon, Go Wun Kim, Ji-Uk Yoon, Hyeonsoo Park, Kyoung-woon Joung

**Affiliations:** 1 Department of Anesthesia and Pain Medicine, Pusan National University Yangsan Hospital, Pusan National University School of Medicine, Yangsan 50612, Republic of Korea; 2 Department of Anesthesia and Pain Medicine, CHA Gangnam Medical Center, CHA University College of Medicine, Seoul 06135, Republic of Korea; 3 Department of Anesthesiology and Pain Medicine, Asan Medical Center, University of Ulsan College of Medicine, Seoul 05505, Republic of Korea

**Keywords:** high-flow nasal cannula, facemask oxygenation, monitored anesthesia care, radiofrequency ablation, hepatic neoplasm, oxygenation, procedural sedation

## Abstract

*Background and Objectives*: Percutaneous liver radiofrequency ablation (RFA) under monitored anesthesia care (MAC) carries a risk of hypoxia due to respiratory depression. Ensuring adequate oxygenation during such procedures is essential for patient safety. This study aimed to evaluate whether a high-flow nasal cannula (HFNC) improves oxygenation compared to a simple facemask during RFA. *Materials and Methods*: In this prospective, randomized controlled trial, 51 patients undergoing ultrasound-guided RFA under MAC were allocated to receive oxygen via an HFNC (30 L/min) or a facemask (6 L/min). Arterial blood gases were collected at the baseline and 5 min after oxygenation. The primary outcome was the arterial partial pressure of oxygen (PaO_2_). Secondary outcomes included hypoxia incidence (SpO_2_ < 95%), the difference in the ratio of the arterial partial pressure of oxygen to the fraction of inspired oxygen concentration (ΔP/F ratio), the difference in the arterial partial pressure of carbon dioxide (ΔPaCO_2_), respiratory rate (RR) changes, and patient satisfaction. *Results*: After adjustment for the baseline PaO_2_, the HFNC group showed significantly higher intra-procedural PaO_2_ compared to the facemask group (299 ± 18.6 vs. 194 ± 19.0 mmHg, *p* < 0.001). No significant differences were found in the ΔP/F ratio, ΔPaCO_2_, or patient satisfaction. Among the secondary outcomes, RR was more stable in the HFNC group throughout the procedure (Group × Time interaction, *p* = 0.003). *Conclusions*: The HFNC significantly improved intra-procedural PaO_2_ during RFA under MAC but did not reduce hypoxia incidence or improve other clinical outcomes compared to facemask oxygenation. The stability of RR observed with the HFNC may reflect a physiological advantage, though further studies are needed to determine its clinical relevance.

## 1. Introduction

Percutaneous liver radiofrequency ablation (RFA) is an effective, minimally invasive treatment for hepatocellular carcinoma and metastatic liver tumors and offers the benefits of reduced tissue trauma, no radiation exposure, and faster recovery compared to surgery [1,2,3]. However, RFA is frequently painful and requires deep sedation under monitored anesthesia care (MAC), which combines local anesthesia, sedatives, and opioids [4,5]. Sedatives and opioids administered during MAC reduce central respiratory drive and impair protective airway reflexes, which leads to hypoventilation, hypercapnia, and hypoxemia; moreover, pharyngeal muscle relaxation may result in upper airway obstruction, and thereby exacerbate desaturation [6,7] A leading cause of morbidity and mortality during procedural sedation, desaturation-related cardiopulmonary complications have incidences of up to 21.6 per 1000 cases in therapeutic procedures, such as endoscopic retrograde cholangiopancreatography, and mortality rates of up to 0.3 per 1000 cases in gastrointestinal endoscopy [8,9]. Therefore, to maintain patient safety and procedural success, advanced oxygen delivery systems are crucial to minimize the sedation-associated risks of hypoventilation and desaturation.

Conventional oxygen delivery modalities, such as nasal cannulas and simple facemasks, have a limited ability to provide consistent oxygenation. In contrast, the high-flow nasal cannula (HFNC) delivers heated, humidified oxygen at high flow rates, offers high inspired oxygen fractions (FiO_2_), and produces a mild positive end-expiratory pressure (PEEP)-like effect [10,11]. The HFNC has demonstrated benefits in reducing hypoxic events during procedural sedation in various interventions, including gastrointestinal endoscopy, bronchoscopy, and interventional radiology [12,13,14]. The physiological advantages of the HFNC may be particularly valuable during painful procedures such as liver RFA, where the depth of sedation may unpredictably deepen and increase the risk of respiratory compromise. However, the HFNC’s efficacy in liver RFA under MAC remains insufficiently elucidated.

This study aimed to evaluate whether, compared to a conventional facemask, an HFNC improves arterial oxygenation, as assessed by the intra-procedural partial pressure of arterial oxygen (PaO_2_), during liver RFA under MAC. We hypothesized that the HFNC, compared to the conventional facemask, would improve arterial oxygenation during liver RFA under MAC. Secondary outcomes included the incidence of hypoxia, changes in the ratio of the arterial partial pressure of oxygen to the fraction of inspired oxygen concentration (∆P/F ratio), changes in the arterial partial pressure of carbon dioxide (∆PaCO_2_), respiratory rate (RR), and patient satisfaction.

## 2. Materials and Methods

### 2.1. Participants

This prospective, randomized, controlled study was approved by the Institutional Review Board of Asan Medical Center (approval number: 2021-0714, 13 May 2021) and registered with the Clinical Research Information Service (registration number: KCT0006221, 17 May 2021). Written informed consent was obtained from all patients prior to enrollment. The study was conducted in accordance with the principles of the Declaration of Helsinki.

Patients aged 20 to 80 years with an American Society of Anesthesiologists (ASA) Physical Status of I or II who were scheduled for ultrasound-guided percutaneous RFA under MAC between July and November 2021 were enrolled. The exclusion criteria included severe chronic pulmonary, cardiovascular, or cerebrovascular disease; a negative modified Allen test; and contraindications to remifentanil or propofol.

Eligible participants were randomized 1:1 to either the HFNC or facemask group using computer-generated block randomization (block size = 2) and sealed opaque envelopes. On the day of the procedure, an investigator (A) who was blinded to outcomes assigned the intervention. A separate investigator (B), who was also blinded, conducted all post-procedural assessments.

### 2.2. Anesthetic Management

All patients underwent continuous monitoring, including non-invasive blood pressure, electrocardiography, peripheral oxygen saturation (SpO_2_), bispectral index (BIS), and RR. RR data were automatically captured by the monitoring system using impedance pneumography and analyzed without further calculation. The monitored hemodynamic parameters were recorded at predefined timepoints (T0, T1, and T2): T0 was defined as the pre-procedural baseline before oxygen delivery; T1 was defined as the timepoint 5 min after the initiation of oxygen administration and sedation during spontaneous breathing; and T2 was defined as the timepoint at the end of the procedure. Following confirmation of collateral circulation with a modified Allen’s test, a 20-G radial arterial catheter was inserted for arterial blood sampling.

Baseline arterial blood gas analysis (ABGA; T0) was performed before oxygen delivery. In the facemask group, 100% oxygen was delivered at 6 L/min via a simple facemask, which approximates an FiO_2_ of 0.5. In the HFNC group, 100% heated and humidified oxygen was delivered at a flow rate of 30 L/min using an OptiFlow system (Fisher & Paykel^®^, Auckland, New Zealand).

Sedation was maintained with a target-controlled infusion of propofol (Marsh model, effect site concentration 0.8–1.5 μg/mL) and remifentanil (starting dose: 0.5 ng/mL, and titrated as needed). Regarding sedation depth, we targeted a BIS of 60–80 and a Modified Observer’s Assessment of Alertness/Sedation (MOAA/S) score ≤ 3 [15]. ABGA was repeated 5 min after oxygen delivery (T1). When the SpO_2_ decreased to <95%, a triple airway maneuver (head tilt, jaw thrust, and mouth opening) was applied, as oxygen reserves rapidly deplete at SpO_2_ levels < 94%, which confers the risk of a swift drop to <90% [16]. If the SpO_2_ further decreased to <90% despite intervention, an airway device was inserted and manual ventilation initiated. All patients were transferred to the post-anesthesia care unit (PACU) for post-procedural monitoring.

### 2.3. Ablation Procedure

After planning sonography, either percutaneous RFA or microwave ablation (MWA) was performed. Artificial ascites (500–1000 mL of 5% dextrose) were induced as needed to enhance ultrasonographic visualization by separating the liver from adjacent structures and to reduce the risk of thermal injury by providing a fluid barrier during ablation. The choice of ablation modality and electrode type (Cool-tip™, Jet-tip^®^, or Proteus^®^) was determined by the interventional radiologist. RFA was conducted using a 200 W generator (Mygen M-2004, RF Medical, Seoul, Republic of Korea) with impedance control for 8 to 18 min. For microwave ablation (MWA), a single 13 G antenna was used with a 150 W generator (Emprint™ HP, Covidien, Minneapolis, MN, USA) for 6–10 min. Additional time was provided for patients with multiple hepatic masses.

### 2.4. Outcome Measurements

The primary outcome of this study was the intra-procedural PaO_2_ that was measured at T1. The PaO_2_ provides a direct, objective measurement of alveolar oxygenation and gas exchange efficiency, and this makes it an appropriate primary outcome for evaluating the effectiveness of oxygen delivery during procedural sedation. Secondary outcomes included the incidence of hypoxia, the ∆P/F ratio and ∆PaCO_2_ between T0 and T1, changes in the RR, and patient satisfaction.

Hypoxia was defined as an SpO_2_ < 95% for procedural safety, and severe hypoxia was defined as an SpO_2_ < 90% despite airway maneuvers and the need for mask ventilation. Based on our institutional protocol to enable early recognition of airway compromise during deep sedation, the 95% threshold was selected. Unlike procedures performed in the lateral or semi-lateral position, percutaneous liver RFA is conducted in the supine position, which significantly increases the risk of glossoptosis. Furthermore, concurrent opioid administration due to severe procedural pain elevates the risk of respiratory depression. Therefore, a conservative SpO_2_ threshold was adopted to identify subtle ventilatory impairment at an early stage and enhance patient safety.

For the calculation of the P/F ratio, the FiO_2_ was assumed to be 1.0 in the HFNC group and 0.5 in the facemask group, based on estimated oxygen delivery via a simple facemask at 6 L/min. Patient satisfaction was assessed using a modified Iowa Satisfaction with Anesthesia Scale (ISAS) [17]. The ISAS comprises 11 items rated on a 6-point scale ranging from −3 to +3, with total scores ranging from −33 to +33. Higher scores indicate greater overall satisfaction. The scale has been validated in monitored anesthesia care and has been applied in procedural sedation settings [18].

### 2.5. Sample Size Calculation

The sample size was calculated using G*Power 3.1 (α = 0.05, power = 0.8), based on a previous study by Heinrich et al., which reported PaO_2_ values as medians (IQR): 406 (362–446) mmHg for the HFNC and 335 (292–389) mmHg for the facemask [19]. These were converted to means ± SD using Meta-Converter (https://meta-converter.com/conversions/mean-sd-iqr, accessed on 5 April 2021), yielding 405 ± 71.3 mmHg and 339 ± 82.3 mmHg, respectively. Based on this, a sample size of 23 per group was required; allowing for a 10% dropout, 26 participants per group (total n = 52) were enrolled.

### 2.6. Statistical Analysis

Continuous variables are presented as the mean ± standard deviation or the median with interquartile range, and categorical variables as frequencies and percentages. Between-group comparisons were performed using Student’s *t*-test or the Mann–Whitney U test for continuous data, and the chi-square test or Fisher’s exact test for categorical data, as appropriate.

To compare the intra-procedural PaO_2_ between the groups, an analysis of covariance (ANCOVA) was performed with the baseline PaO_2_ as a covariate. Similarly, the ΔP/F ratio was adjusted for pre-procedural values using ANCOVA. Linear regression analysis was used to evaluate changes in PaCO_2_, adjusting for potential confounders including age, body mass index (BMI), chronic obstructive pulmonary disease (COPD), asthma, and smoking status. Given the multiple secondary outcomes—including hypoxia incidence, ΔP/F ratio, ΔPaCO_2_, RR, and patient satisfaction—the Bonferroni correction was applied to control for type I errors. For repeated measurements of RR, a generalized linear mixed model (GLMM) with random intercepts was fitted. The model included group, time, and group × time interaction as fixed effects, and subject as a random effect. An unstructured covariance matrix was used without assuming homogeneity across groups. Model assumptions, including the normality of residuals and homogeneity of variance, were assessed for all parametric tests. When assumptions were not fully met, a ranked ANCOVA was performed as a non-parametric alternative to confirm the robustness of the results. As the findings were consistent, the original parametric results were retained for reporting. All statistical analyses were performed using SPSS Statistics version 29.0.2.0 (IBM Corp., Armonk, NY, USA), and a two-sided *p*-value < 0.05 was considered statistically significant.

## 3. Results

**Figure 1:**
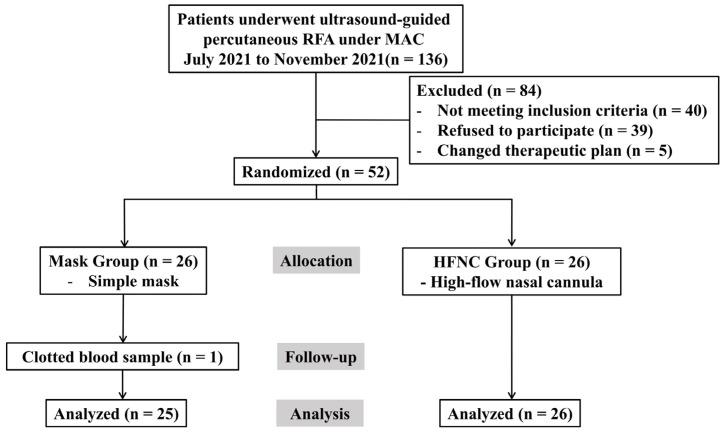
A flow chart of the study inclusion process according to the CONSORT statement. RFA, radiofrequency ablation; MAC, monitored anesthesia care; HFNC, high-flow nasal cannula.

**Figure 2:**
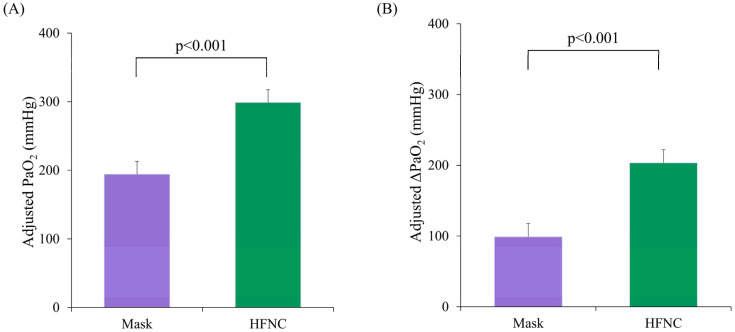
A comparison of the arterial oxygen partial pressure (PaO_2_) between the facemask (Mask) and HFNC groups during liver radiofrequency ablation. (**A**) The intra-procedural adjusted mean PaO_2_ between the Mask and HFNC groups during the procedure, analyzed using analysis of covariance (ANCOVA) with the baseline PaO_2_ as a covariate. (**B**) The changes in PaO_2_ (ΔPaO_2_) from the baseline to intra-procedure between the two groups. The adjusted ΔPaO_2_ values were analyzed using ANCOVA. The error bars represent the standard error of the mean (SE). HFNC, high-flow nasal cannula.

A total of 136 patients scheduled for RFA under MAC between July and November 2021 were screened. Of these, 40 did not meet the inclusion criteria, 39 declined to participate, and 5 were excluded due to changes in their therapeutic plan. Finally, 52 patients were randomized; however, 1 patient in the facemask group was excluded because of clotted blood samples. Therefore, 51 patients (25 and 26 in the facemask and HFNC groups, respectively) were included in the final analysis (Figure 1). The baseline characteristics were comparable between groups (Table 1), despite significantly different pre-procedure PaO_2_ levels (*p* = 0.037). Using ANCOVA with the pre-procedural PaO_2_ as a covariate, the adjusted mean PaO_2_ during the procedure was significantly higher in the HFNC group (298.8 ± 18.6 mmHg; 95% confidence interval [CI]: 261.4–336.2) compared to the facemask group (194.2 ± 19.0 mmHg; 95% CI: 156.0–232.3, *p* < 0.001; Figure 2A). Similarly, the adjusted mean ΔPaO_2_ (T_1_–T_0_) was significantly greater in the HFNC group (203.4 ± 18.6 vs. 98.8 ± 19.0; difference = 104.6, *p* < 0.001; Figure 2B).

**Figure 3:**
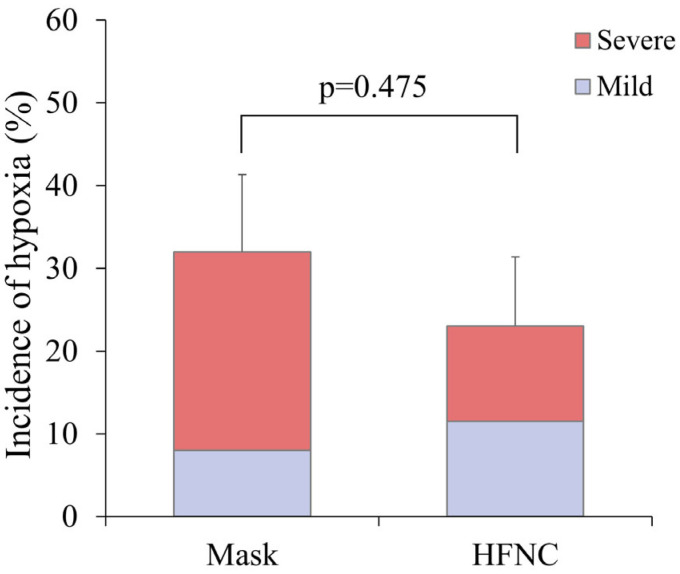
A comparison of the incidence of hypoxia between the facemask (Mask) and HFNC groups during liver radiofrequency ablation. Hypoxia was categorized as mild (SpO_2_ < 95%) or severe (SpO2 < 90% despite triple airway maneuver). The stacked bar graph illustrates the proportion of mild and severe hypoxia in each group. While the incidence of severe hypoxia was higher in the Mask group, the overall incidence of hypoxia did not differ significantly between the two groups (*p* = 0.475).

Hypoxia occurred in 14 patients (27.5%), including 8 (32.0%) in the facemask group and 6 (23.0%) in the HFNC group (*p* = 0.475) (Figure 3). Severe hypoxia occurred more frequently in the facemask group (24.0% vs. 11.5%), corresponding to a risk ratio of 0.48 (95% CI: 0.13–1.72). However, the difference was not statistically significant (*p* = 0.291). Other ABGA parameters, including ΔP/F ratio and ΔPaCO_2_, did not differ significantly between groups (Table 2). Patient satisfaction, as assessed by the ISAS, was also similar between groups (Table 2).

**Figure 4:**
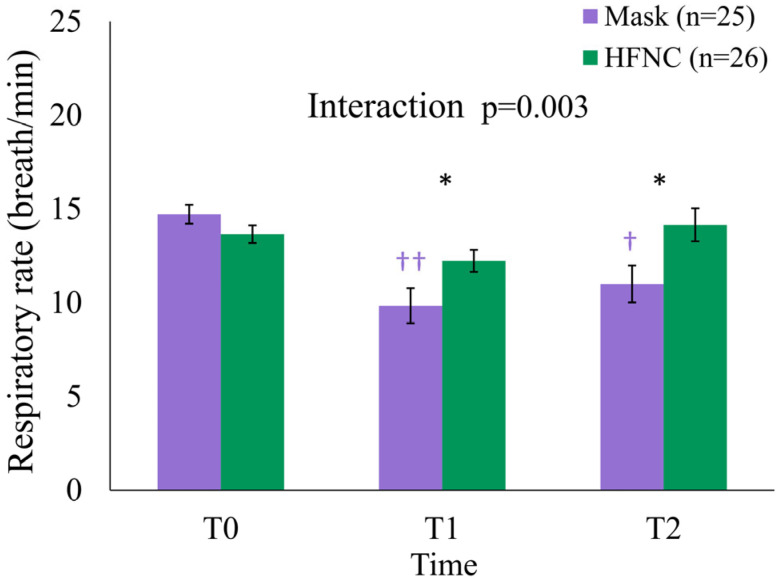
The changes in respiratory rate (RR) over time in the facemask (Mask) and HFNC groups during liver radiofrequency ablation. The data are presented as the mean ± standard error. Significant group-by-time interaction was observed (*p* = 0.003, generalized linear mixed model (GLMM)). Between-group differences were significant at T1 and T2 (* *p* = 0.036 and * *p* = 0.031, respectively). Within-group analysis showed that, in the Mask group, RR decreased at T1 (†† *p* < 0.001) and T2 († *p* = 0.002) compared to T0, with no significant changes in the HFNC group. Symbols: * *p* < 0.05; † *p* < 0.05, †† *p* < 0.001 (within group). HFNC, high-flow nasal cannula; T0, pre-procedure; T1, intra-procedure; T2, post-procedure.

At T0, the mean RR was 13.7 ± 2.4 and 14.7 ± 2.5 breaths/min in the HFNC and facemask groups, respectively (*p* = 0.131) (Table 3). At T1 and T2, the mean RR was significantly higher in the HFNC group (T1: 12.2 ± 3.0 vs. 9.8 ± 4.7 breaths/min, *p* = 0.036; T2: 14.2 ± 4.5 vs. 11.0 ± 4.9 breaths/min, *p* = 0.031). A generalized linear mixed model (GLMM) revealed a significant group × time interaction (*p* = 0.003) and a main effect of time (*p* = 0.024). Within-group analysis demonstrated significant RR reductions in the facemask group from T0 to T1 (*p* < 0.001) and from T0 to T2 (*p* = 0.002), whereas RR in the HFNC group remained stable (Table 3, Figure 4).

## 4. Discussion

This randomized controlled trial demonstrated that the HFNC significantly improved intra-procedural arterial oxygenation compared to a simple facemask during percutaneous liver RFA under MAC. The marked difference in ΔPaO_2_ highlights the dynamic effect of the HFNC in enhancing alveolar oxygenation beyond baseline levels. However, despite this physiological improvement, the incidence of hypoxia (SpO_2_ < 95%) did not differ significantly between groups, raising questions about the clinical translation of improved oxygenation.

The lack of difference in hypoxia incidence likely reflects the multifactorial nature of oxygen desaturation during deep sedation. Notably, our institutional protocol mandated predefined interventions when SpO_2_ dropped below 95%, which included a triple airway maneuver to rapidly restore oxygen saturation. These early responses may have mitigated desaturation severity and reduced between-group differences. In fact, most desaturation events resolved promptly without escalation to invasive support; only two patients required manual ventilation, and none required intubation. Taken together, these observations imply that upper airway obstruction, rather than inadequate oxygen delivery, may be a dominant cause of hypoxia during MAC. Glossoptosis induced by sedation, especially in the supine position with opioid co-administration, can precipitate airway collapse and compromise ventilation despite high FiO_2_ delivery [20,21]. This is consistent with previous observations that the HFNC improves oxygenation but may not prevent desaturation when upper airway patency is lost [6,7].

Although the HFNC theoretically offers continuous positive airway pressure (PEEP)-like effects through high flow rates, the flow setting of 30 L/min in our study was likely insufficient to generate clinically meaningful airway pressure. Prior studies indicate that flow rates ≥ 40–60 L/min are required to produce measurable PEEP and facilitate alveolar recruitment [22,23]. However, higher flows were not feasible during RFA due to patient discomfort and interference with procedural accuracy. Therefore, while this moderate flow setting reflects real-world procedural constraints, it may have limited the physiological potential of the HFNC. Still, the significant PaO_2_ improvement observed in the HFNC group suggests enhanced oxygenation even without optimal PEEP. Another consideration is the role of gas reabsorption atelectasis associated with high FiO_2_ delivery. Deep sedation and supine positioning predispose patients to alveolar collapse, and the administration of 100% oxygen via the HFNC may exacerbate this through nitrogen washout and resorption. This phenomenon may counteract the benefits of increased FiO_2_ and help explain the unchanged incidence of hypoxia [24].

Although the observed difference in severe hypoxia (11.5% in the HFNC group vs. 24.0% in the facemask group) corresponds to a risk ratio of 0.48, the wide 95% CI (0.13–1.72) reflects substantial uncertainty. While not statistically significant, the trend toward reduced severe hypoxia in the HFNC group may be clinically relevant and warrants further evaluation in larger, adequately powered studies.

These findings suggest that the physiological improvements observed with the HFNC may have potential clinical relevance in high-risk patients, such as those with impaired pulmonary function, obesity, or obstructive sleep apnea, where even modest improvements in oxygenation and respiratory stability could help prevent desaturation events and respiratory compromise. Further studies that focus on high-risk populations are needed to confirm the definitive impact of the HFNC on patient prognosis.

Moreover, the FiO_2_ delivered via the HFNC is theoretically fixed but may be diluted by ambient air due to mouth breathing or irregular respiratory patterns, particularly under sedation. Studies have shown that FiO_2_ dilution increases with open-mouth breathing and high inspiratory flow demands, potentially limiting effective oxygen delivery [22,25]. In our study, although we assumed FiO_2_ values of 1.0 (HFNC) and 0.5 (facemask) for P/F ratio calculations, these estimates are subject to variability and should be interpreted cautiously.

Interestingly, RR remained more stable in the HFNC group throughout the procedure. This contrasts with the facemask group, which exhibited a reduction in RR that persisted post-procedurally. A stable RR is clinically meaningful, as irregular or suppressed respiratory patterns may lead to hypoventilation and hypoxemia. While sedation level and opioid dosing were equivalent across groups, the observed difference may reflect the physiological benefits of the HFNC, such as reduced inspiratory workload and improved comfort [26,27,28].

Despite this, we observed no significant difference in arterial PaCO_2_ levels between groups. This aligns with previous reports in settings such as bronchoscopy, thoracic surgery, and COPD care, where the HFNC had minimal impact on PaCO_2_ due to its limited effect on minute ventilation [14,16,25,29,30,31,32]. While the HFNC can flush out nasopharyngeal dead space and aid CO_2_ elimination, deep sedation-induced hypoventilation likely blunts its effectiveness in this context [26,31]. Patient satisfaction, assessed using the validated ISAS, was similarly high in both groups, suggesting that the method of oxygen delivery did not negatively affect patient experience under deep sedation. The lack of difference in satisfaction may be explained by several factors. Although previous studies have demonstrated improved comfort with the HFNC compared to non-invasive ventilation or other oxygen delivery methods [33,34], the large-bore nasal cannula may have caused mild discomfort in our patients. Moreover, the relatively short procedural duration and the use of deep sedation likely reduced patients’ awareness of external stimuli, making it difficult for them to perceive differences between oxygen delivery methods. These factors, in combination, may have contributed to the absence of a measurable difference in satisfaction between the groups. Future studies may explore the potential impact of the HFNC on procedural success and workflow efficiency, such as reducing airway interventions or procedural interruptions. In addition, cost effectiveness analyses that consider the prevention of post-procedural complications and recovery-related healthcare costs may further support the practical value of the HFNC in clinical practice.

This study has several limitations. First, the small sample size was powered only for the primary outcome (PaO_2_) and not for secondary outcomes such as hypoxia, ΔP/F ratio, ΔPaCO_2_, or satisfaction. A post hoc power analysis indicated that the statistical power for these variables was insufficient (e.g., 11.1% for hypoxia, 5.1% for ΔPaCO_2_), introducing the possibility of a type II error and limiting definitive conclusions. Further multicenter studies with larger sample sizes are warranted to validate these findings and to optimize clinical protocols. Second, ABGA was collected at a fixed intra-procedural timepoint rather than during actual desaturation events. This limits its utility in assessing real-time respiratory compromise and weakens its correlation with SpO_2_-based hypoxia. Future studies should consider transcutaneous gas monitoring or real-time ABG sampling during desaturation episodes to better capture dynamic gas exchange. Lastly, although we assumed an FiO_2_ of 0.5 for the facemask and 1.0 for the HFNC group based on conventional estimates, interindividual variability in FiO_2_ delivery remains a limitation. Our study aimed to evaluate whether the HFNC, which generally provides higher and more consistent FiO_2_, improves oxygenation compared to the standard oxygen therapy commonly used during deep sedation. However, given the practical limitations of HFNC application during liver RFA, future studies comparing the HFNC with other high-flow oxygen delivery methods, such as non-rebreather masks, may help to identify more effective alternatives.

Despite these limitations, this study addresses oxygenation strategies during sedation with combined sedatives and opioids for painful therapeutic procedures such as liver RFA. The deeper sedation level and supine positioning increase the risk of upper airway obstruction due to glossoptosis and hypoventilation, which heighten the risk of hypoxemia. These high-risk conditions have been less extensively examined in prior studies on oxygenation during procedural sedation. Our findings suggest that although the HFNC improves oxygenation, careful selection of flow rates is needed to balance oxygenation and procedural feasibility in this setting.

## 5. Conclusions

In conclusion, the HFNC improved intra-procedural arterial oxygenation and stabilized RR in patients undergoing liver RFA under MAC, despite the lack of observed differences in clinical endpoints such as hypoxia incidence or patient satisfaction. These findings support the physiological utility of the HFNC in this setting and highlight the need for further studies examining optimized flow rates, individualized FiO_2_ delivery, and patient subgroups at higher risk of desaturation.

## Figures and Tables

**Table 1:** The baseline characteristics and clinical variables.

	Mask (n = 25)	HFNC (n = 26)	*p*-Value
Age (y)	61.1 ± 8.5	63.2 ± 7.9	0.360
Sex (male/female)	21 (84.0)/4 (16.0)	23 (88.5)/3 (11.5)	0.703
BMI (kg/m^2^)	24.6 ± 3.6	24.9 ± 3.1	0.762
Diabetes	7 (28.0)	10 (38.5)	0.428
Hypertension	9 (36.0)	9 (34.6)	0.918
COPD	4 (16.0)	2 (7.7)	0.419
ASA classification			0.110
I	3 (12.0)	0(0.0)	
II	22 (88.0)	26 (100.0)	
Tumor type			1.000
HCC	24 (96.0)	23 (88.5)	
Metastatic	1 (4.0)	2 (7.7)	
Cryptogenic LC	0 (0.0)	1 (3.8)	
Tumor size (cm)	1.6 ± 0.5	1.5 ± 0.4	0.310
Pre-procedure ABGA			
pH (mmHg)	7.4 (7.4, 7.4)	7.4 (7.4, 7.4)	0.572
PaCO_2_ (mmHg)	38.3 (35.6, 40.4)	37.5 (31.8, 39.0)	0.158
PaO_2_ (mmHg)	85.8 (77.9, 98.8)	95.4 (86.2, 107.0)	0.037
HCO_3_^−^ (mmol/l)	24.3 ± 2.6	23.2 ± 3.0	0.173
P/F ratio	171.6 (155.7, 197.6)	95.4 (86.2 107.0)	<0.001
Total ablation time (min)	8.0 (6.0, 10.0)	6.5 (5.0, 12.0)	0.374
Sedation time (min)	34.0 (29.5, 47.5)	28.0 (26.0, 40.5)	0.262
Total dose of propofol (mg)	77.0 (67.0, 103.0)	75.0 (57.1, 99.0)	0.445
Total dose of remifentanil (ng)	88.7 (70.6, 108.0)	76.1 (63.5, 96.3)	0.366
Type of procedure			0.301
Conventional	17 (68.0)	14 (53.8)	
Microwave	8 (32.0)	12 (46.2)	
Use of artificial ascites	7 (28.0)	7 (26.9)	0.931

The data are expressed as the mean ± standard deviation, median (interquartile range), or number of patients (percentage), as appropriate. HFNC, high-flow nasal cannula; BMI, body mass index; COPD, chronic obstructive pulmonary disease; ASA, American Society of Anesthesiologists; HCC, hepatocellular carcinoma; LC, liver cirrhosis; ABGA, arterial blood gas analysis; PaCO_2_, partial pressure of arterial carbon dioxide; PaO_2_, partial pressure of arterial oxygen; P/F ratio, the ratio of the partial pressure of arterial oxygen to the fraction of inspired oxygen concentration.

**Table 2:** A comparison of the adjusted outcomes and patient satisfaction between the Mask and HFNC groups.

	Mask (n = 25)	HFNC (n = 26)	Crude *p*-Value	Bonferroni- Corrected *p*-Value
Incidence of hypoxia (95% CI)	32.0 ± 9.3 (13.7–50.3)	23.1 ± 8.3 (6.9–39.3)	0.475	1.000
Intra-procedure ABGA				
∆P/F ratio (95% CI) *	246.1 ± 30.8 (184.2−308.0)	158.6 ± 30.0 (98.3−218.9)	0.084	0.336
∆PaCO_2_ (mmHg) (95% CI) †	13.6 ± 4.59 (4.30–22.8)	14.2 ± 5.00 (4.13–24.3)	0.789	1.000
Satisfaction				
Patient satisfaction (95% CI)	25.0 ± 2.1 (20.7–29.4)	21.1 ± 2.4 (16.1–26.1)	0.270	1.000

All intra-procedure ABGA measurements were adjusted for baseline differences using analysis of covariance (ANCOVA). The adjusted values are expressed as the mean ± standard error (95% confidence interval). * Adjusted for pre-procedure P/F ratio. † Adjusted for body mass index, age, COPD, asthma, and smoking status. Patient satisfaction is presented as the median (interquartile range). HFNC, high-flow nasal cannula; ABGA, arterial blood gas analysis; ∆P/F ratio, the difference in the ratio of the partial pressure of arterial oxygen to the fraction of inspired oxygen concentration between T1 and T0; ∆PaCO_2_, the difference between the partial pressure levels of arterial carbon dioxide measured at T1 and T0. COPD, chronic obstructive pulmonary disease; T0, pre-procedure; T1, intra-procedure; CI, confidence interval.

**Table 3:** Time-course changes in respiratory rate (breath/min).

Variable		Group			Analysis for Repeated Measures	
	Average (n = 51)	Mask (n = 25)	HFNC (n = 26)	*p*	Source	*p* *
Respiratory rate (breath/min)						
T0	14.2 ± 2.5	14.7 ± 2.5 ^a^	13.7 ± 2.4 ^a^	0.131 ^1^	Group	0.090
T1	11.1 ± 4.1	9.8 ± 4.7 ^b^	12.2 ± 3.0 ^a^	0.036 ^1^	Time	0.024
T2	12.6 ± 4.9	11.0 ± 4.9 ^b^	14.2 ± 4.5 ^a^	0.031 ^2^	Group × Time	0.003

Values are expressed as the mean ± standard deviation (SD). Bonferroni’s post hoc test was used for multiple comparisons between timepoints. Superscript letters (a, b) within the same group indicate statistically significant differences between timepoints (*p* < 0.05). Means sharing the same letter are not significantly different. ^1^ *p* values were derived from independent *t*-tests. ^2^ *p* values were derived from the Mann–Whitney *U* test. * *p* values were derived from the Generalized Linear Mixed Model. The Shapiro–Wilk test was employed to test normality assumptions.

## Data Availability

The datasets used and analyzed in the current study are available from the corresponding author upon reasonable request.

## Abbreviations

The following abbreviations are used in this manuscript:

RFA	Radiofrequency ablation
MAC	Monitored anesthesia care
HFNC	High-flow nasal cannula
FiO_2_	Fraction of inspired oxygen
PEEP	Positive end-expiratory pressure
SpO_2_	Peripheral oxygen saturation
PaO_2_	Arterial oxygen partial pressure
PaCO_2_	Arterial partial pressure of carbon dioxide
RR	Respiratory rate
BIS	Bispectral index
ABGA	Arterial blood gas analysis
P/F	Ratio of arterial partial pressure of oxygen to fraction of inspired oxygen
ISAS	Iowa Satisfaction with Anesthesia Scale
ANCOVA	Analysis of covariance
COPD	Chronic obstructive pulmonary disease
CI	Confidence interval
